# Gefitinib initiates sterile inflammation by promoting IL-1β and HMGB1 release via two distinct mechanisms

**DOI:** 10.1038/s41419-020-03335-7

**Published:** 2021-01-06

**Authors:** Takuya Noguchi, Yuto Sekiguchi, Yuki Kudoh, Rio Naganuma, Tomohiro Kagi, Akiko Nishidate, Kazuhiro Maeda, Chizuru Ishii, Takashi Toyama, Yusuke Hirata, Gi-Wook Hwang, Atsushi Matsuzawa

**Affiliations:** 1 Laboratory of Health Chemistry, Graduate School of Pharmaceutical Sciences, Tohoku University, Sendai, Japan; 2 Laboratory of Molecular and Biochemical Toxicology, Graduate School of Pharmaceutical Sciences, Tohoku University, Sendai, Japan; 3 Laboratory of Environmental and Health Sciences, Faculty of Pharmaceutical Sciences, Tohoku Medical and Pharmaceutical University, Sendai, Japan

**Keywords:** Inflammasome, Inflammatory diseases

## Abstract

Anticancer drug gefitinib causes inflammation-based side effects, such as interstitial pneumonitis. However, its mechanisms remain unknown. Here, we provide evidence that gefitinib elicits pro-inflammatory responses by promoting mature-interleukin-1β (IL-1β) and high-mobility group box 1 (HMGB1) release. Mitochondrial reactive oxygen species (mtROS) driven by gefitinib stimulated the formation of the NLRP3 (NACHT, LRR and PYD-containing protein 3) inflammasome, leading to mature-IL-1β release. Notably, gefitinib also stimulated HMGB1 release, which is, however, not mediated by the NLRP3 inflammasome. On the other hand, gefitinib-driven mtROS promoted the accumulation of γH2AX, a hallmark of DNA damage, leading to the activation of poly (ADP-ribose) polymerase-1 (PARP-1) and subsequent active release of HMGB1. Together our results reveal the potential ability of gefitinib to initiate sterile inflammation via two distinct mechanisms, and identified IL-1β and HMGB1 as key determinants of gefitinib-induced inflammation that may provide insights into gefitinib-induced interstitial pneumonitis.

## Introduction

NLRP3/cryopyrin is a member of the NOD-like receptor (NLR) protein family that plays a pivotal role in the inflammatory response against pathogens and cellular damage. It does so by forming a multiprotein signaling complex, the NLRP3 inflammasome, with apoptosis-associated speck-like protein containing CARD (ASC) and caspase-1^1^. A wide variety of inflammatory mediators such as pathogen-associated molecular patterns (PAMPs) and damage-associated molecular patterns (DAMPs) trigger the formation and activation of the NLRP3 inflammasome. The NLRP3 then processes the precursor pro-IL-1β into mature-IL-1β and triggers pro-inflammatory cell death termed pyroptosis^2^. Excessive or prolonged activation of the NLRP3 inflammasome has been implicated in the pathogenesis of diverse diseases, including arteriosclerosis, gout, type II diabetes mellitus, and Alzheimer disease^1,2^. Moreover, gain-of-function mutations in NLRP3 can cause the cryopyrin-associated periodic syndrome (CAPS), resulting in an aberrant oversecretion of IL-1β due to spontaneous activation of NLRP3 inflammasome^3^. Thus, the NLRP3 inflammasome is considered an attractive therapeutic target to improve the inflammatory diseases associated with IL-1β oversecretion, yet the regulatory mechanisms are not fully understood.

To date, several mechanisms of the NLRP3 inflammasome activation have been proposed, and particularly its relevance to mitochondrial dynamics has been demonstrated^4–6^. One such proposed mechanism is that major NLRP3 ligands such as pore-forming toxins, extracellular ATP, and microcrystals, induce the loss of mitochondrial membrane potential (MMP) that leads to the release of DAMPs such as mitochondrial reactive oxidative species (mtROS) and mitochondrial DNA (mtDNA), which in turn activate the NLRP3 inflammasome^4,7^. Another such mechanism is that a number of NLRP3 ligands elicit mitochondrial damage arising from Ca^2+^ overload, which triggers NLRP3 inflammasome activation^4,8^. However, mechanisms by which damaged mitochondria activates the NLRP3 inflammasome are still highly debated^4^.

High-mobility group box 1 (HMGB1) is a nuclear protein that binds to DNA and acts as an architectural chromatin-binding factor, although its roles are not fully understood^9^. Additionally, HMGB1 has emerged as an extracellular danger signal that mediates the pro-inflammatory responses, and in particular, triggers the activation of NLRP3 and AIM2 inflammasomes^10^. It has been demonstrated that HMGB1 can be both passively and actively released from necrotic and activated cells, respectively^9^. Although mechanisms that involve the active release of HMGB1 are largely unknown, previous studies have demonstrated the involvement of poly (ADP-ribose) polymerase-1 (PARP-1)^11,12^. PARP-1 mediates poly-ADP-ribosylation (pADPr, or PARylation), which is a ubiquitous post-translational modification that modulates various cellular processes, including gene expression, DNA repair, and protein function and location just as pADPr of HMGB1 accelerates its extracellular release^11^. Thus, emerging evidence suggests an important role of PARP-1 as an inflammatory mediator.

Pneumonitis is a general term that refers to the inflammation of lung tissue caused by drugs, inflammatory agents, and infection. It has been reported that lung tissues of patients with pneumonitis exhibited increased production of pro-inflammatory cytokines, such as TNF-α, IL-1β, and IL-18^13,14^. Moreover, drug-induced pneumonitis has been particularly implicated in the inflammasome-mediated IL-1β and IL-18 production. For instance, pneumonitis induced by bleomycin, a glycopeptide anticancer agent, is mitigated in IL-18 or caspase-1 knockout mice^15^. Meanwhile, the expression levels of NLRP3, ASC, and caspase-1 are elevated in lung tissue damaged by rituximab, an anti-CD20 monoclonal antibody approved for non-Hodgkin lymphoma^16^. These findings indicate the existence of mechanisms by which these anticancer agents activate the inflammasome. However, these mechanisms have not been well studied.

Epidermal growth factor receptor (EGFR) is a cell surface receptor tyrosine kinase that transduces growth signals^17^. Overexpression or mutations of EGFR that promotes tumorigenesis have been found in many different types of cancers, and thus have been highlighted its role as an attractive therapeutic target^18,19^. Gefitinib is a molecular target drug that specifically blocks the tyrosine kinase activity of EGFR through preventing its autophosphorylation, and is approved for the treatment for patients with advanced non-small cell lung cancer (NSCLC)^20–22^. However, it has been reported that gefitinib unexpectedly targets unexpected proteins besides EGFR, and causes diffuse alveolar damage associated with acute interstitial pneumonia^21,23–25^. In previous study using rat, gefitinib was shown to promote the production of IL-1β, leading to exacerbation of acute lung injury, suggesting that gefitinib has an ability to activate the inflammasome, which may be responsible, at least in part, for gefitinib-induced pneumonitis^26^. However, the underlying mechanism remains unknown.

In the present study, to determine whether gefitinib initiates sterile inflammation at a cellular level, and if so, to uncover the molecular mechanisms, we examined the effects of gefitinib on macrophages, and found that gefitinib promotes the mature-IL-1β and HMGB1 release in macrophages. Interestingly, gefitinib-driven reactive oxidative species (ROS) elicit the activation of the NLRP3 inflammasome and PARP-1, which promotes IL-1β and HMGB1 release, respectively. Thus, our results demonstrate the possibility that gefitinib initiate sterile inflammation via at least two distinct mechanisms, which may be crucial for understanding the pathogenesis of gefitinib-induced pneumonitis.

## Results

### Gefitinib promotes mature-IL-1β release in macrophages

**Fig. 1:**
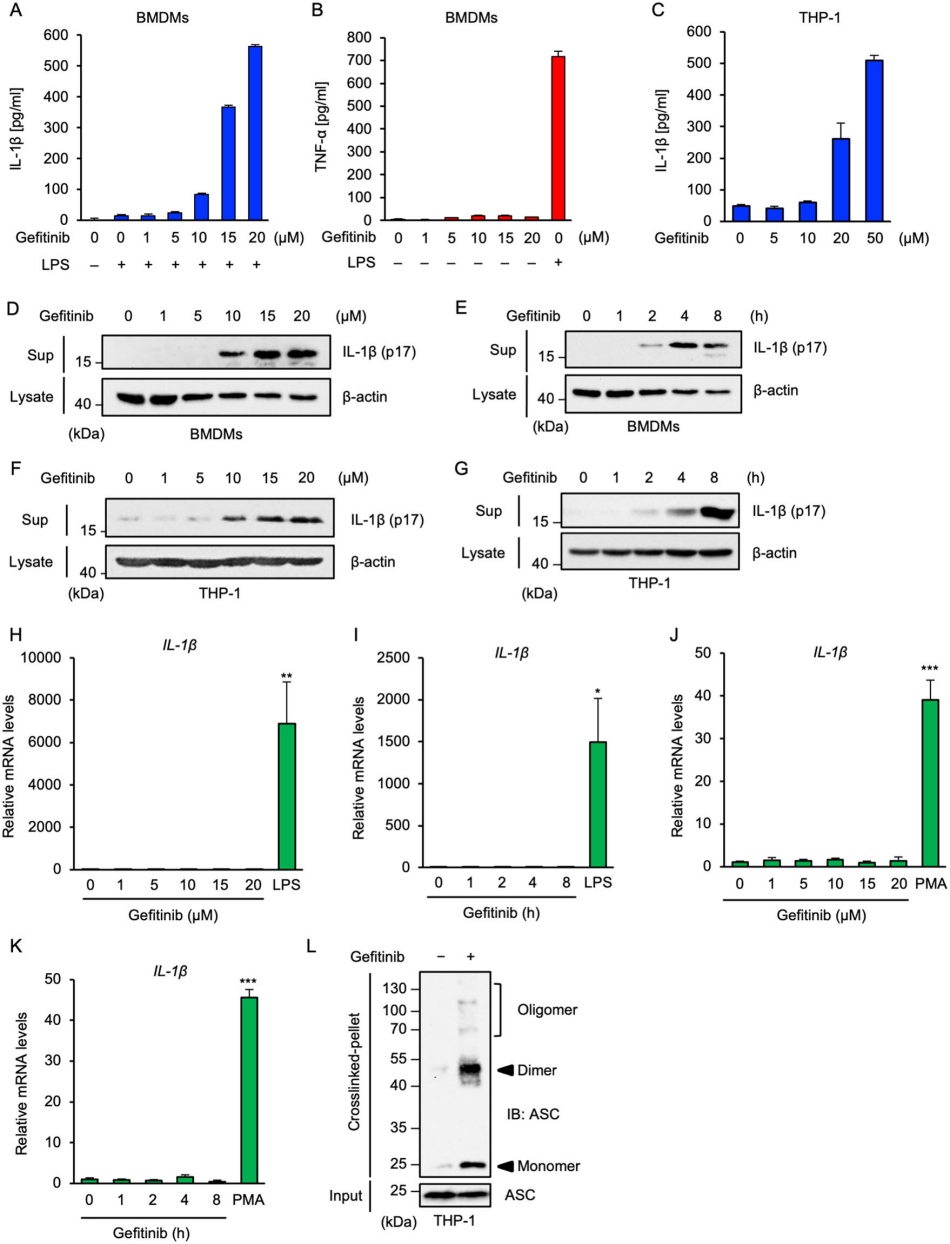
Gefitinib promotes mature-IL-1β release in macrophages. **A**, **B** ELISA analysis of cytokines in culture supernatants of BMDMs. LPS-primed or unprimed BMDMs were treated with the indicated concentrations of gefitinib for 8 h. IL-1β (**A**) or TNF-α (**B**) release were analyzed by ELISA. **C** ELISA analysis of cytokines in culture supernatants of THP-1 cells. PMA-differentiated THP-1 cells were treated with the indicated concentrations of gefitinib for 8 h. IL-1β release was analyzed by ELISA. **D**, **E** Immunoblot analysis of IL-1β in culture supernatants of BMDMs. LPS-primed BMDMs were treated with the indicated concentrations of gefitinib for 8 h (**D**), or treated with 20 μM gefitinib for indicated periods (**E**). Cell-free supernatants (Sup) and cell lysates were subjected to immunoblotting with the indicated antibodies. **F**, **G** Immunoblot analysis of IL-1β in culture supernatants of THP-1 cells. PMA-differentiated THP-1 cells were treated with the indicated concentrations of gefitinib for 8 h (**F**), or treated with 20 μM gefitinib for the indicated periods (**G**). Cell-free supernatants (Sup) and cell lysates were subjected to immunoblotting with the indicated antibodies. **H**, **I** The mRNA expression levels of IL-1β. BMDMs were treated with the indicated concentrations of gefitinib for 8 h or 100 ng/ml LPS for 6 h (**H**), or treated with 20 μM gefitinib for indicated periods or 100 ng/ml LPS for 6 h (**I**). The mRNA levels of *IL-1β* were analyzed by quantitative real-time PCR (normalized with *GAPDH* mRNA levels). Graphs are shown as mean ± S.D. (*n* = 3). Statistical significance was determined by student’s *t*-test; *p < 0.05, **p < 0.01. **J**, **K** The mRNA expression levels of IL-1β. THP-1 cells were treated with the indicated concentrations of gefitinib for 8 h or 100 nM PMA for 4 h (**J**), or treated with 20 μM gefitinib for indicated periods or 100 nM PMA for 4 h (**K**). The mRNA levels of *IL-1β* were analyzed by quantitative real-time PCR (normalized with *GAPDH* mRNA levels). Graphs are shown as mean ± S.D. (*n* = 3). Statistical significance was determined by student’s *t*-test; ***p < 0.001. **L** ASC oligomerisation assay in THP-1 cells. PMA-differentiated THP-1 cells were treated with 20 μM gefitinib for 8 h. DSS-mediated crosslinked pellets (crosslinked-pellets) and soluble lysates (Input) were subjected to immunoblotting with anti-ASC antibody. All data in Fig. 1 are representatives of at least three independent experiments.

To elucidate the molecular basis of inflammation-associated adverse reactions induced by gefitinib, we focused on the cellular responses of macrophages. Results from an enzyme-linked immunosorbent assay (ELISA) revealed that when gefitinib was used to treat mouse bone marrow-derived macrophages (BMDMs) primed with lipopolysaccharides (LPS), a toll-like receptor 4 (TLR4) ligand that transcriptionally upregulates pro-IL-1β expression, gefitinib promotes the production of IL-1β (Fig. 1A). However, gefitinib failed to stimulate TNF-α production, whereas LPS could strongly do so (Fig. 1B). In addition, gefitinib also promoted the production of IL-1β in human monocytic THP-1 cells that were differentiated into macrophages by phorbol myristate acetate (PMA) (Fig. 1C). An immunoblot analysis confirmed that gefitinib promotes mature-IL-1β release in a concentration- and time-dependent manner in BMDMs (Fig. 1D, E) and PMA-primed THP-1 cells (Fig. 1F, G). We next examined whether gefitinib affects pro-IL-1β expression at transcriptional level, because transcriptional upregulation of pro-IL-1β is an essential process for the mature-IL-1β release^1^. However, we found that gefitinib failed to upregulate pro-IL-1β at mRNA levels both in BMDMs (Fig. 1H, I) and THP-1 cells (Fig. 1J, K), whereas LPS or PMA strongly upregulated. These observations suggest that gefitinib promotes the release of mature-IL-1β by activating post-transcriptional steps. Given that gefitinib stimulates mature-IL-1β release, it is reasonable to assume that gefitinib has the ability to activate the inflammasomes that govern the post-transcriptional steps of IL-1β release. It is known that ASC oligomerisation is a hallmark of the inflammasome activation, and its detection by immunoblot analysis has been well established^27,28^. We thus performed an ASC oligomerisation assay and found that gefitinib clearly induces ASC oligomerisation, suggesting that gefitinib promotes mature-IL-1β release by activating the inflammasomes (Fig. 1L).

### Gefitinib promotes IL-1β release by stimulating the NLRP3 inflammasome

**Fig. 2:**
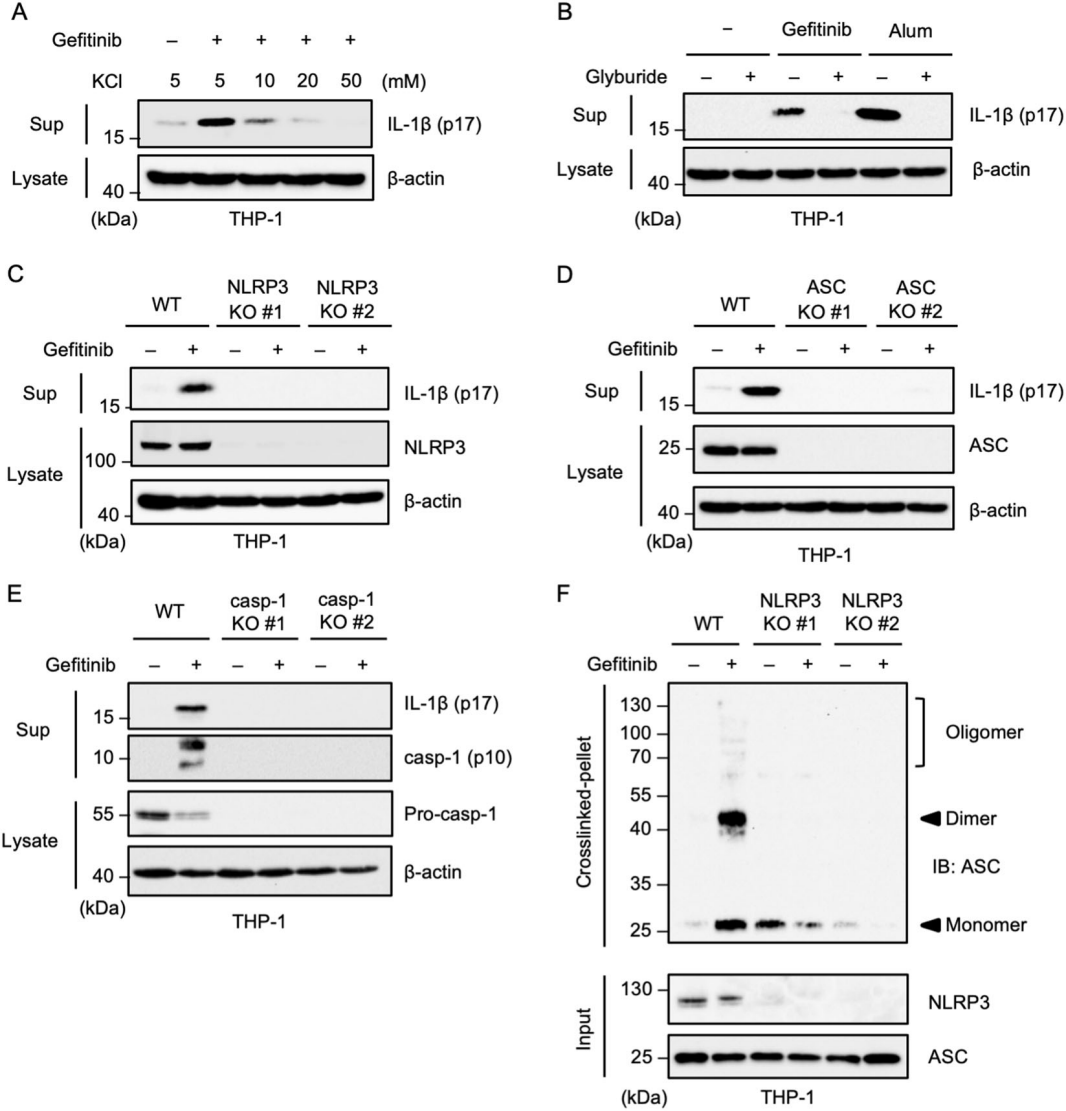
Gefitinib promotes IL-1β release by stimulating the NLRP3 inflammasome. **A** The inhibitory effect of KCl on gefitinib-induced IL-1β release. PMA-differentiated THP-1 cells were treated with 20 μM gefitinib for 8 h in the presence of the indicated concentrations of KCl. Cell-free supernatants (Sup) and cell lysates were subjected to immunoblotting with the indicated antibodies. **B** The inhibitory effect of glyburide on gefitinib-induced IL-1β release. PMA-differentiated THP-1 cells were pretreated with 100 μM glyburide for 0.5 h and then treated with 20 μM gefitinib for 8 h or 200 µg/ml Alum for 6 h. Cell-free supernatants (Sup) and cell lysates were subjected to immunoblotting with the indicated antibodies. **C**, **D**, **E** Requirement of the NLRP3 inflammasome for gefitinib-induced IL-1β release. PMA-differentiated WT, NLRP3 KO, ASC KO, and caspase-1 KO THP-1 cells were treated with 20 μM gefitinib for 8 h. Cell-free supernatants (Sup) and cell lysates were subjected to immunoblotting with the indicated antibodies. **F** ASC oligomerisation assay in NLRP3 KO THP-1 cells. PMA-differentiated WT and NLRP3 KO THP-1 cells were treated with 20 μM gefitinib for 8 h. DSS-mediated crosslinked pellets (crosslinked-pellet) and soluble lysates (Input) were subjected to immunoblotting with the indicated antibodies. All data in Fig. 2 are representatives of at least five independent experiments.

We then examined which of the inflammasomes were activated by gefitinib. Interestingly, we found that the release of gefitinib-induced IL-1β was strongly inhibited by the typical NLRP3 inflammasome inhibitors, such as KCl (Fig. 2A) and glyburide (also called glibenclamide), as well as alum that is widely used as a NLRP3 ligand (Fig. 2B)^29,30^. These observations prompted us to determine whether the NLRP3 inflammasome was involved in the gefitinib-induced IL-1β release. To this end, we analyzed NLRP3 knockout (KO) THP-1 cells generated by using the Clustered Regularly Interspaced Short Palindromic Repeat/CRISPR-associated protein-9 nuclease (CRISPR/Cas9) system as previously described^31^. As expected, gefitinib-induced IL-1β release was strongly attenuated in NLRP3 KO THP-1 cells (Fig. 2C). Knockout of ASC or caspase-1 was also found to suppress gefitinib-induced IL-1β release (Fig. 2D, E). Moreover, the ASC oligomerisation is completely suspended in NLRP3 KO THP-1 cells (Fig. 2F). Collectively, these observations strongly indicate that gefitinib promotes IL-1β release by activating the NLRP3 inflammasome.

### Gefitinib elicits mitochondrial damage and subsequent mtROS generation

**Fig. 3:**
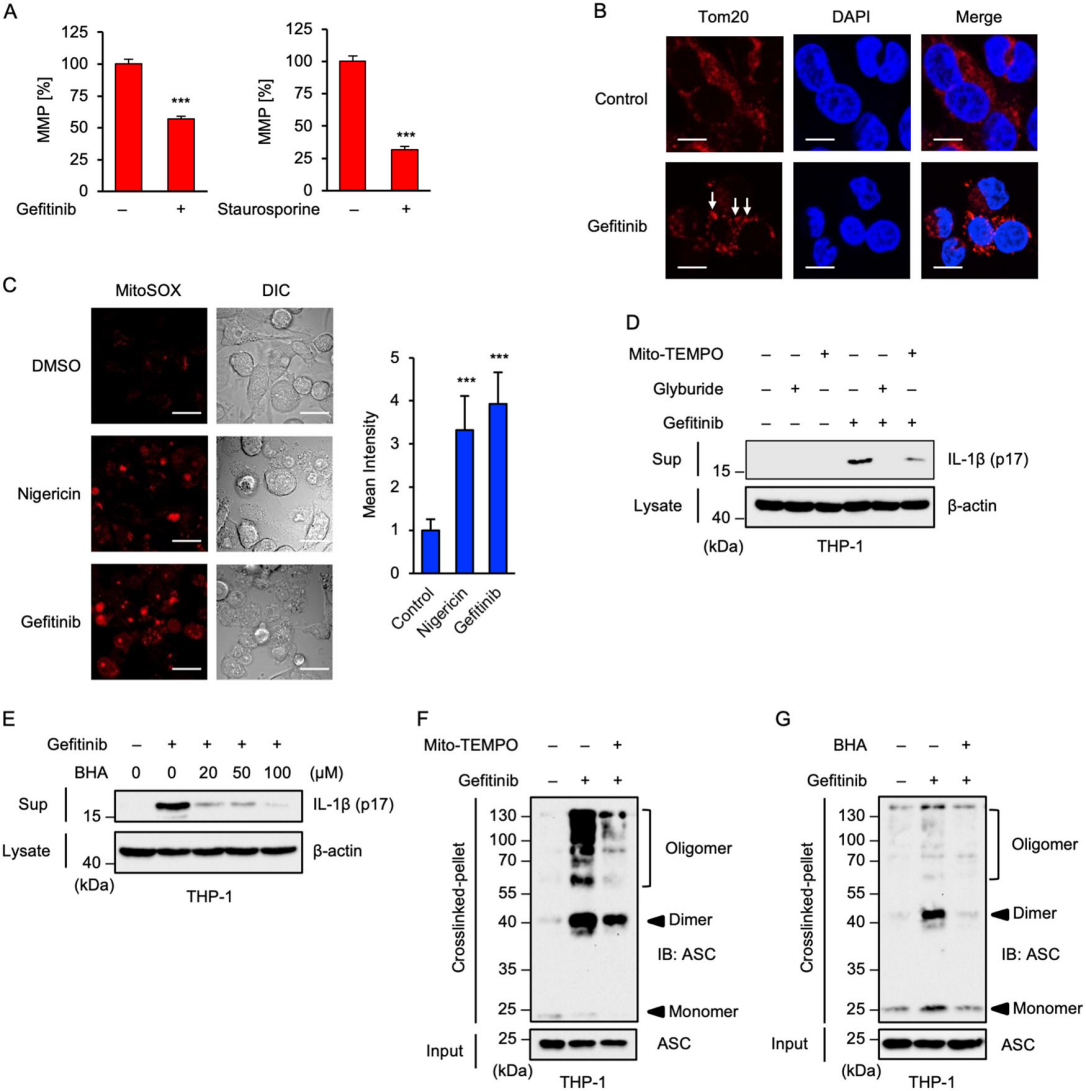
Gefitinib elicits mitochondrial damage and subsequent mtROS generation. **A** JC-1 assay to detect loss of mitochondrial membrane potential (MMP) in THP-1 cells. PMA-differentiated THP-1 cells were treated with 20 μM gefitinib or 1 μM Staurosporin for 8 h. MMP was measured using JC-1 probe. Data shown are the mean ± S.D. Significant differences were determined by student’s *t*-test; ***p < 0.001. **B** Immunofluorescence imaging of mitochondrial aggregation in gefitinib-treated THP-1 cells. PMA-differentiated THP-1 cells were treated with 20 μM gefitinib for 8 h, and then performed immunofluorescence staining with Tom20 antibody, and 4’,6-diamidino-2-phenylindole (DAPI) nuclear staining (Scale bar, 10 μm). Arrows indicate the aggregated mitochondria. All images are representatives of three independent experiments. **C** Immunofluorescence imaging of mtROS generation in gefitinib-treated THP-1 cells. PMA-differentiated THP-1 cells were treated with 20 μM gefitinib for 8 h, and then stained with MitoSOX. Fluorescence images and intensity were acquired as described in the materials and methods section. Cell morphology was determined by Nomarski differential interference contrast (DIC) microscopy (Scale bar, 20 μm). All images are representatives of three independent experiments and graphs are shown as the mean ± S.D. Significant differences were determined by student’s *t*-test; ***p < 0.001. **D** The inhibitory effect of Mito-TEMPO on gefitinib-induced IL-1β release. PMA-differentiated THP-1 cells were pretreated with 200 μM Mito-TEMPO or 100 μM glyburide for 0.5 h and then treated with 20 μM gefitinib for 8 h. Cell-free supernatants (Sup) and cell lysates were subjected to immunoblotting with the indicated antibodies. **E** The inhibitory effect of BHA on gefitinib-induced IL-1β release. PMA-differentiated THP-1 cells were pretreated with the indicated concentrations of BHA for 0.5 h and then treated with 20 μM gefitinib for 8 h. Cell-free supernatants (Sup) and cell lysates were subjected to immunoblotting with the indicated antibodies. **F**, **G** ASC oligomerisation assay in THP-1 cells. PMA-differentiated THP-1 cells were pretreated with 200 μM Mito-TEMPO (**F**) or 100 μM BHA (**G**) for 0.5 h and then treated with 20 μM gefitinib for 8 h. DSS-mediated crosslinked pellets (Crosslinked-pellet) and soluble lysates (Input) were subjected to immunoblotting with anti-ASC antibody. All data and images in Fig. 3 are representatives of at least three independent experiments.

Next, we explored the mechanisms by which gefitinib activates the NLRP3 inflammasome. Recent studies have demonstrated that mitochondria can play a pivotal role for mitochondria in the NLRP3 inflammasome activation^4,32^. In particular, loss of MMP due to mitochondrial damage allows the release of DAMPs, such as mtROS, which can lead to NLRP3 inflammasome activation^4^. Moreover, a previous report has shown that gefitinib causes mitochondrial dysfunction^33^. Thus, by using a fluorescent probe 5, 5’, 6, 6’-tetrachloro-1, 1’, 3, 3’-tetraethylbenzimidazolyl-carbocyanine iodide (JC-1), we examined whether gefitinib affected MMP^34^. As shown in Fig. 3A, gefitinib clearly reduced MMP, although to a lesser extent than the protein kinase inhibitor staurosporine that is widely used as a positive control for JC-1 assay. Moreover, we found that gefitinib causes mitochondrial aggregation, which is observed when mitochondria are damaged (Fig. 3B)^35,36^. These observations thus suggest that gefitinib causes mitochondrial damage. Since leakage of DAMPs, including mtROS, caused by mitochondrial damage triggers NLRP3 inflammasome activation, we examined whether gefitinib stimulates generation of mtROS^4,32^. Bioimaging using the mtROS-specific indicator MitoSOX clearly showed that mtROS generation was induced by gefitinib and nigericin, a typical ligand of the NLRP3 inflammasome (Fig. 3C). Co-treatment with antioxidants, such as the mitochondria-targeted antioxidant Mito-TEMPO (Fig. 3D) and butylated hydroxyanisole (BHA) (Fig. 3E), strongly suppressed gefitinib-induced mature-IL-1β release. Moreover, both antioxidants prevented ASC oligomerisation (Fig. 3F, G). Collectively, these observations suggest that gefitinib-driven mtROS are responsible for the NLRP3 inflammasome activation.

### Gefitinib-driven mtROS cause pyroptosis

**Fig. 4:**
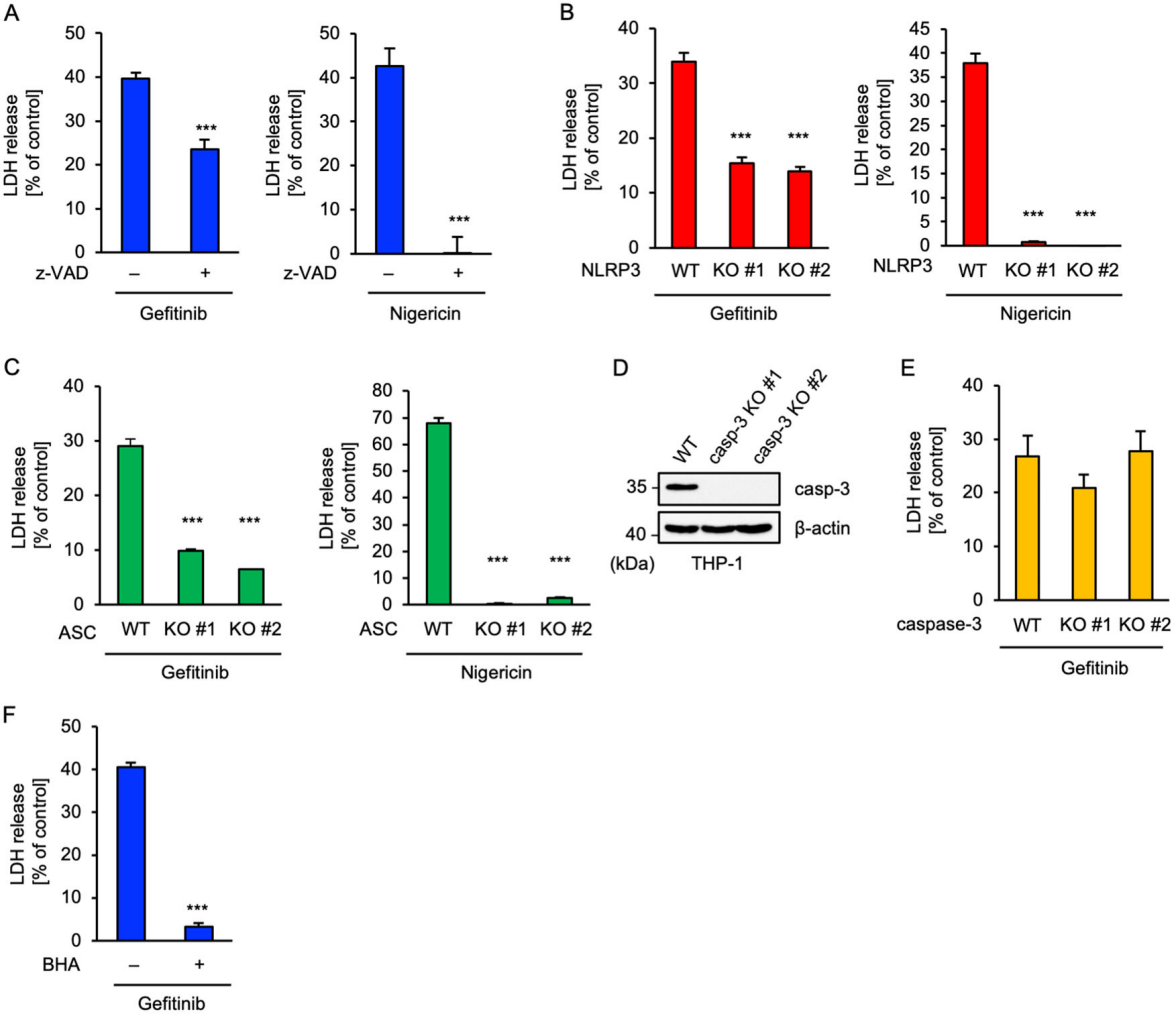
Gefitinib-driven mtROS cause pyroptosis. **A** The inhibitory effect of z-VAD on gefitinib-induced cell death. PMA-differentiated THP-1 cells were pretreated with 20 μM z-VAD for 0.5 h and then treated with 20 μM gefitinib for 8 h or 5 μM nigericin for 2 h. Cell cytotoxicity was measured by LDH release assay. Data shown are the mean ± S.D. Significant differences were determined by student’s *t*-test; ***p < 0.001. **B**, **C** Requirement of NLRP3 or ASC for gefitinib-induced cell death. PMA-differentiated WT and NLRP3 KO (**B**) or ASC KO (**C**) THP-1 cells were treated with 20 μM gefitinib for 8 h or 5 μM nigericin for 2 h. Cell cytotoxicity was measured by LDH release assay. Data shown are the mean ± S.D. Significant differences were determined by student’s *t*-test; ***p < 0.001. **D** Immunoblot analysis of caspase-3 in THP-1 cells. Whole-cell lysates were subjected to immunoblotting with the indicated antibodies. **E** Requirement of caspase-3 for gefitinib-induced cell death. PMA-differentiated WT and caspase-3 KO THP-1 cells were treated with 20 μM gefitinib for 8 h. Cell cytotoxicity was measured by LDH release assay and cell lysates were subjected to immunoblotting with the indicated antibodies. Data shown are the mean ± S.D. **F** The inhibitory effect of BHA on gefitinib-induced cell death. PMA-differentiated THP-1 cells were pretreated with 100 μM BHA for 0.5 h and then treated with 20 μM gefitinib for 8 h. Cell cytotoxicity was measured by LDH release assay. Data shown are the mean ± S.D. Significant differences were determined by student’s *t*-test; ***p < 0.001. All data in Fig. 4 are representatives of at least five independent experiments.

Activation of the NLRP3 inflammasome mediates not only mature-IL-1β release but also a novel type of caspase-1-dependent inflammatory cell death, termed pyroptosis^37,38^. Pyroptosis is different from apoptosis in that it is accompanied by the release of immunogenic cellular content, including mature-IL-1β, which accelerates inflammation^37,38^. We therefore investigated the involvement of pyroptosis in gefitinib-induced inflammation. Lactate dehydrogenase (LDH) assay revealed that gefitinib-induced death of macrophages was significantly suppressed by the pan-caspase inhibitor z-VAD-fmk, although to a lesser extent than nigericin (Fig. 4A). Consistent with these findings, both NLRP3 and ASC KO THP-1 cells exhibited significant resistance to gefitinib-induced cell death, though not as much as nigericin (Fig. 4B, C). Therefore, these results indicated that pyroptosis might be involved in gefitinib-induced inflammation. Next, we investigated the involvement of apoptosis in gefitinib-induced macrophage death, since previous reports have demonstrated gefitinib-induced apoptosis in other cell types^39,40^. To this end, we established two independent clones of caspase-3 KO THP-1 cells with the knowledge that caspase-3 is the main executor of apoptosis (Fig. 4D), and found that caspase-3 KO THP-1 cells are not resistant to gefitinib-induced cell death (Fig. 4E). This showed that the contribution of apoptosis mediated by caspase-3 to gefitinib-induced cell death in macrophages is relatively small compared with pyroptosis. Additionally, it was found that BHA largely suppressed gefitinib-induced cell death (Fig. 4F). Thus, in accord with the NLRP3 activation, gefitinib-induced pyroptosis is most likely to be mediated by mtROS-driven oxidative stress.

### Gefitinib promotes HMGB1 release independently of the NLRP3 inflammasome

**Fig. 5:**
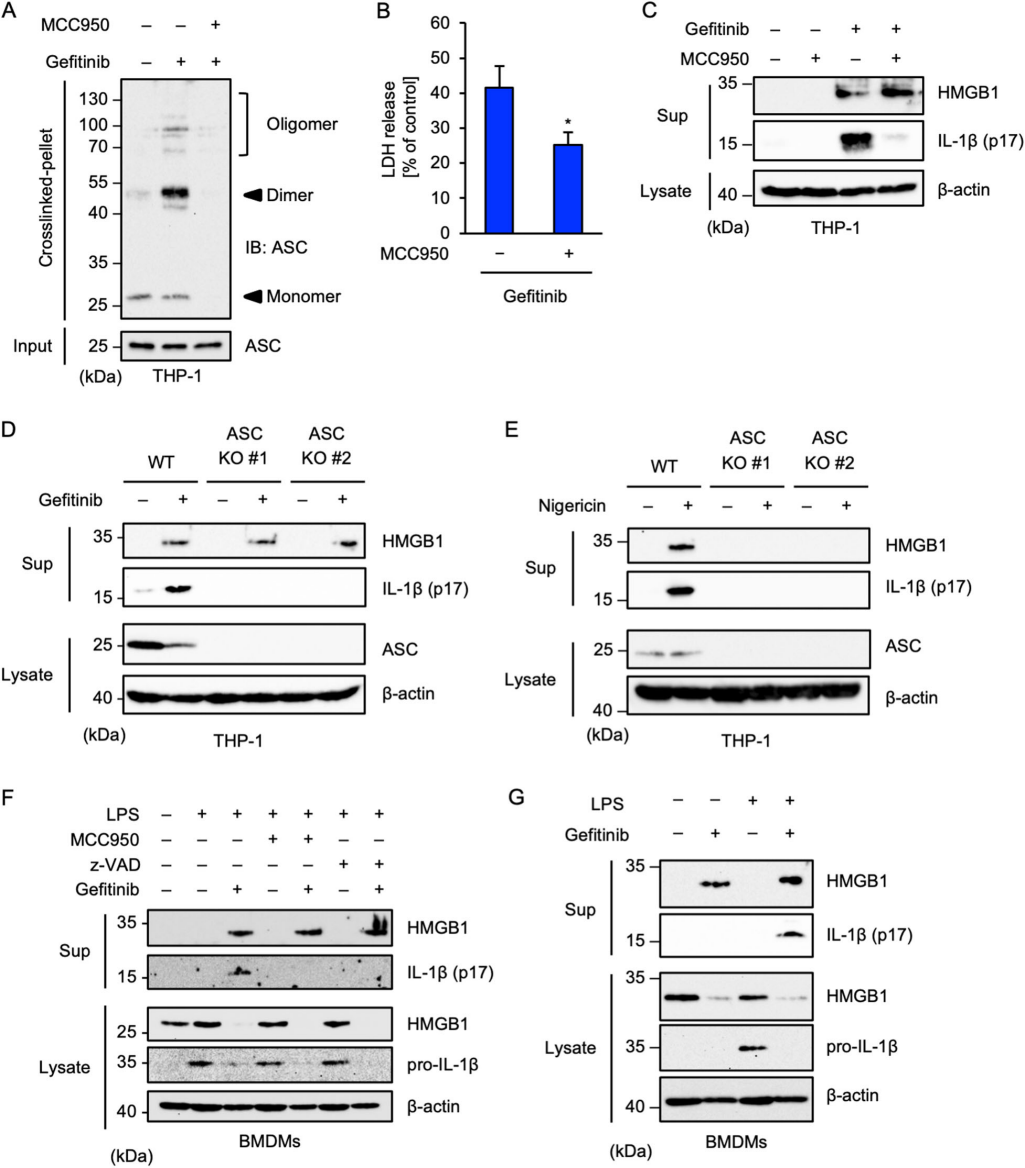
Gefitinib promotes HMGB1 release independently of the NLRP3 inflammasome. **A**, **B**, **C** The inhibitory effect of MCC950. PMA-differentiated THP-1 cells were pretreated with 1 μM MCC950 for 0.5 h and then treated with 20 μM gefitinib for 8 h. **A** DSS-mediated crosslinked pellets (Crosslinked-pellet) and soluble lysates (Input) were immunoblotted with anti-ASC antibody. **B** Cell cytotoxicity was measured by LDH release assay. Data shown are the mean ± S.D. Significant differences were determined by student’s *t*-test; *p < 0.05. **C** Cell-free supernatants (Sup) and cell lysates were subjected to immunoblotting with the indicated antibodies. **D**, **E** Requirement of ASC in gefitinib- or nigericin-induced HMGB1 release. PMA-differentiated WT and ASC KO THP-1 cells were treated with 20 μM gefitinib for 8 h or 5 μM nigericin for 2 h. Cell-free supernatants (Sup) and cell lysates were subjected to immunoblotting with the indicated antibodies. **F** The inhibitory effect of MCC950 or z-VAD on gefitinib-induced HMGB1 release. LPS-primed BMDMs were pretreated with 1 μM MCC950 or 20 µM z-VAD for 0.5 h and then treated with 20 μM gefitinib for 8 h. Cell-free supernatants (Sup) and cell lysates were subjected to immunoblotting with the indicated antibodies. **G** Requirement of LPS priming for gefitinib-induced HMGB1 release. LPS-primed or unprimed BMDMs were treated with 20 μM gefitinib for 8 h. Cell-free supernatants (Sup) and cell lysates were subjected to immunoblotting with indicated antibodies. All data in Fig. 5 are representatives of at least three independent experiments.

Activation of the NLRP3 inflammasome stimulates not only mature-IL-1β but also HMGB1 extracellular release and extracellular HMGB1 is known to activate the NLRP3 inflammasome by binding its receptors, including RAGE (receptor for advanced glycation endproducts) and TLR2/4^9,41,42^. HMGB1 release facilitates a positive feedback loop of the NLRP3 inflammasome activation, which triggers excessive inflammation. On the other hand, recent evidence has suggested the involvement of HMGB1 in pneumonitis^43,44^. We therefore examined the involvement of HMGB1 in specifically gefitinib-induced inflammation. As shown in Fig. 5A, MCC950, a specific NLRP3 inhibitor identified recently^45^, completely suppressed the ASC oligomerisation induced by gefitinib. Moreover, MCC950 inhibited gefitinib-induced cell death to a similar extent as z-VAD-fmk did, as shown in Fig. 4A, and strongly inhibited mature-IL-1β release strongly (Fig. 5B, C). These observations support the idea that gefitinib activates the NLRP3 inflammasome and demonstrates the effectiveness of MCC950 as a NLRP3 inhibitor. Interestingly, we found that gefitinib stimulates HMGB1 release, which is, however, not inhibited by MCC950 (Fig. 5C). Gefitinib-induced HMGB1 release was not attenuated in ASC knockout THP-1 cells (Fig. 5D), whereas nigericin-induced HMGB1 and mature-IL-1β release was completely attenuated, as well as mature-IL-1β release (Fig. 5E). Therefore, the involvement of the NLRP3 inflammasome in HMGB1 release appears to be context-dependent. We also investigated gefitinib-induced HMGB1 release in BMDMs. As shown in Fig. 5F, both MCC950 and z-VAD-fmk could inhibit gefitinib-induced mature-IL-1β but not HMGB1 release in BMDMs. Notably, gefitinib clearly promoted HMGB1 release without LPS priming (Fig. 5G), meaning that TLR4-dependent transcriptional upregulation is dispensable for gefitinib-induced HMGB1 release. Together, these findings indicate that gefitinib promotes HMGB1 release through other mechanisms rather than the NLRP3 inflammasome.

### Gefitinib-induced HMGB1 release is mediated by PARP-1 activation

**Fig. 6:**
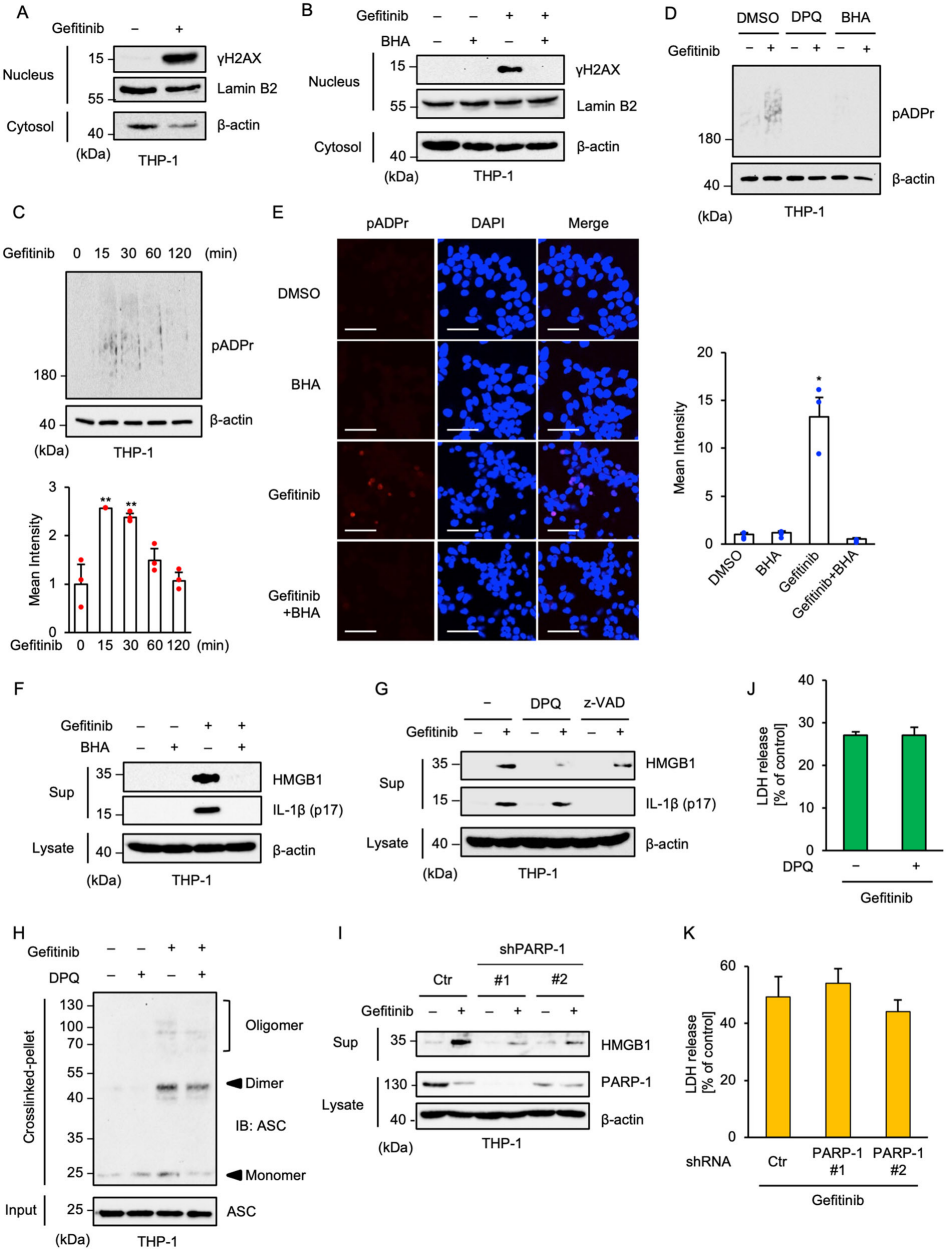
Gefitinib-induced HMGB1 release is mediated by PARP-1 activation. **A** Accumulation of γH2AX in gefitinib-treated THP-1 cells. PMA-differentiated THP-1 cells were treated with 20 μM gefitinib for 8 h. The nuclear and cytoplasmic extracts were subjected to immunoblotting with the indicated antibodies. **B** The inhibitory effect of BHA on the γH2AX accumulation. PMA-differentiated THP-1 cells were pretreated with the 100 µM BHA for 0.5 h and then treated with 20 μM gefitinib for 8 h. The nuclear and cytoplasmic extracts were subjected to immunoblotting with the indicated antibodies. **C** Enhancement of poly-ADP-ribosylation (pADPr) in gefitinib-treated THP-1 cells. PMA-differentiated THP-1 cells were treated with 20 μM gefitinib for the indicated periods. Whole-cell extracts were subjected to immunoblotting with the indicated antibodies. Graphs depict the value of means and S.D. of three independent experiments. Significant differences were determined by student’s *t*-test; **p < 0.01. **D** The inhibitory effect of DPQ and BHA on gefitinib-induced PARP-1 activation. PMA-differentiated THP-1 cells were pretreated with 30 μM DPQ or 100 µM BHA for 0.5 h and then treated with 20 μM gefitinib for 1 h. Whole-cell extracts were subjected to immunoblotting with the indicated antibodies. **E** The inhibitory effect of BHA on gefitinib-induced PARP-1 activation. PMA-differentiated THP-1 cells were pretreated with the 100 µM BHA for 0.5 h and then treated with 20 μM gefitinib for 8 h, and then performed immunofluorescence staining with pADPr antibody, and 4’,6-diamidino-2-phenylindole (DAPI) nuclear staining (Scale bar, 50 μm). All images are representatives of three independent experiments and graphs depict the value of means and S.D. of three independent fields. Significant differences were determined by student’s *t*-test; *p < 0.05. **F** The inhibitory effect of BHA on gefitinib-induced HMGB1 release. PMA-differentiated THP-1 cells were pretreated with 100 μM BHA for 0.5 h and then treated with 20 μM gefitinib for 8 h. Cell-free supernatants (Sup) and cell lysates were subjected to immunoblotting with the indicated antibodies. **G** The inhibitory effect of DPQ on gefitinib-induced HMGB1 release. PMA-differentiated THP-1 cells were pretreated with 30 μM DPQ or 20 μM z-VAD for 0.5 h and then treated with 20 μM gefitinib for 8 h. Cell-free supernatants (Sup) and cell lysates were subjected to immunoblotting with the indicated antibodies. **H** The inhibitory effect of DPQ on gefitinib-induced ASC oligomerisation. PMA-differentiated THP-1 cells were pretreated with 30 μM DPQ for 0.5 h and then treated with 20 μM gefitinib for 8 h. DSS-mediated crosslinked pellets (Crosslinked-pellet) and soluble lysates (Input) were immunoblotted with anti-ASC antibodies. **I** Requirement of PARP-1 for gefitinib-induced HMGB1 release. PMA-differentiated PARP-1 KD THP-1 cells were treated with 20 μM gefitinib for 8 h. Cell-free supernatants (Sup) and cell lysates were subjected to immunoblotting with the indicated antibodies. **J** The inhibitory effect of DPQ on gefitinib-induced cell death. PMA-differentiated THP-1 cells were pretreated with 30 μM DPQ for 0.5 h and then treated with 20 μM gefitinib for 8 h. Cell cytotoxicity was measured by LDH release assay. Data shown are the mean ± S.D. **K** Gefitinib-induced cell death in PARP-1 KD cells. PMA-differentiated PARP-1 KD THP-1 cells were treated with 20 μM gefitinib for 8 h. Cell cytotoxicity was measured by LDH release assay. Data shown are the mean ± S.D. All data and images in Fig. 6 are representatives of at least three independent experiments.

In steady state, HMGB1 localizes in the nucleus, and works as a DNA-binding protein that regulates gene expression and DNA replication^46^. Under stress conditions, HMGB1 is exported to the cytoplasm, and then secreted into the extracellular space^12^. Several reports have demonstrated that the export mechanisms of HMGB1 are controlled by nuclear effectors, including PARP-1^11,12^. Thus, we focused on the nuclear events caused by gefitinib and found the nuclear accumulation of γH2AX, an indicator of DNA damage, in gefitinib-treated cells (Fig. 6A). Moreover, the accumulation of γH2AX was clearly attenuated by co-treatment with BHA, suggesting that oxidative stress driven by gefitinib-induced mtROS accumulation elicits DNA damage (Fig. 6B). PARP-1 recognizes DNA damage, and coordinates DNA repair by promoting poly-ADP-ribosylation (pADPr) of the components involved in the DNA repair machinery^47^. Therefore, it is known that pADPr of the nuclear proteins is an index of PARP-1 activation. We observed gefitinib-induced pADPr as smear bands (Fig. 6C). In addition, the smear bands were attenuated by the co-treatment with the PARP-1 inhibitor 3, 3’, 5, 5’-tetra-tert-butyldiphenoquinone (DPQ) or BHA (Fig. 6D). Moreover, immuno-fluorescent staining of pADPr revealed that gefitinib particularly promoted pADPr of nuclear proteins, which is suppressed by co-treatment with BHA (Fig. 6E). These observations suggest that gefitinib causes PARP-1 activation through oxidative stress-induced DNA damage. We then examined the involvement of PARP-1 in gefitinib-induced HMGB1 release. Similarly to the PARP-1 activation as shown in Fig. 6D, gefitinib-induced HMGB1 release was strongly attenuated by co-treatment with BHA (Fig. 6F). Moreover, co-treatment with DPQ clearly suppressed gefitinib-induced HMGB1, whereas DPQ failed to suppress gefitinib-induced mature-IL-1β release (Fig. 6G) and ASC oligomerisation (Fig. 6H). On the contrary, z-VAD-fmk failed to suppress gefitinib-induced HMGB1 release, whereas mature-IL-1β release was completely attenuated (Fig. 6G), supporting the idea that gefitinib-induced HMGB1 release is not mediated by the NLRP3 inflammasome. Consistent with these results, we found that gefitinib-induced HMGB1 release is also reduced in PARP-1 knockdown THP-1 cells (Fig. 6I). Meanwhile, both DPQ treatment and PARP-1 knockdown did not affect gefitinib-induced cell death (Fig. 6J, K). Collectively, these results suggest that the release of both HMGB1 and mature-IL-1β depend on gefitinib-induced ROS generation with each release being mediated by distinct mechanisms.

### Gefitinib activates the NLRP3 inflammasome in vivo

**Fig. 7:**
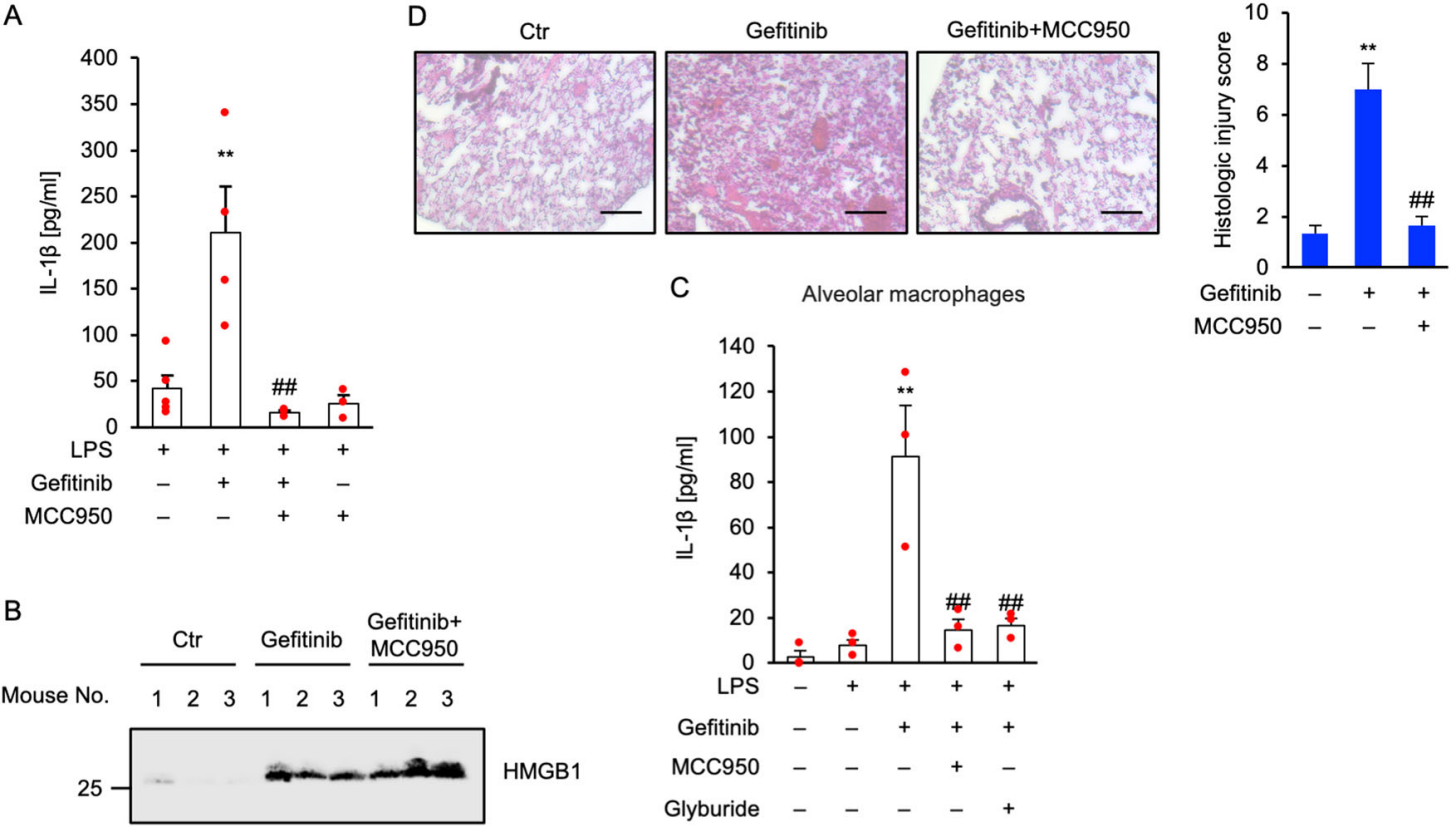
Gefitinib activates the NLRP3 inflammasome in vivo. **A**, **B** The inhibitory effect of MCC950 on gefitinib-induced IL-1β or HMGB1 release in vivo. **A** Mice were treated with 1 µg LPS and 20 mg/kg MCC950 by intraperitoneal injection for 2 h, and then treated with 10 mg/kg gefitinib with 20 mg/kg MCC950 by intraperitoneal injection. After 1 h, peritoneal lavage fluid was collected, and IL-1β levels were analyzed by ELISA. Graphs are shown as mean ± SEM (LPS: *n* = 5, LPS + gefitinib: *n* = 4, LPS + gefitinib + MCC950: *n* = 3, LPS + MCC950: *n* = 3). Statistical significance was determined by one-way ANOVA, followed by Tukey–Kramer test; **p < 0.01 (versus LPS), ##*p* < 0.01 (versus LPS + gefitinib). **B** Mice were treated with 25 mg/kg MCC950 by intraperitoneal for 0.5 h, and then treated with 10 mg/kg gefitinib by intraperitoneal injection. After 0.5 h, 25 mg/kg MCC950 was reinjected. After 4 h, peritoneal lavage fluid was collected, and were subjected to immunoblotting with anti-HMGB1 antibody. **C** The inhibitory effect of MCC950 or glyburide on gefitinib-induced IL-1β release in alveolar macrophages. LPS-primed alveolar macrophages isolated from C57BL/6 mice were pretreated with 1 µM MCC950 or 100 µM glyburide for 0.5 h and then treated with 20 µM gefitinib for 24 h. IL-1β levels in culture supernatants were analyzed by ELISA. Graphs are shown as mean ± SEM (*n* = 3). Statistical significance was determined by one-way ANOVA, followed by Tukey–Kramer test; **p < 0.01 (versus LPS), ##*p* < 0.01 (versus LPS + gefitinib). **D** The inhibitory effect of MCC950 on gefitinib-induced acute lung injury. Mice were treated with 20 mg/kg gefitinib and 2.5 mg/kg MCC950 by oropharyngeal aspiration. After 48 h, lung tissues were stained with HE staining (scale bar, 50 μm), and histologic injury scores were calculated as described in the materials and methods section. Graphs are shown as mean ± SEM for three different fields. Statistical significance was determined by one-way ANOVA, followed by Tukey–Kramer test; **p < 0.01 (versus Ctr: Control), ##*p* < 0.01 (versus Gefitinib). All data in Fig. 7 are representatives of at least three independent experiments.

We provided evidence that gefitinib initiates mature-IL-1β and HMGB1 release via distinct mechanisms at the cellular level. In turn, we tested whether gefitinib can activate both mechanisms in vivo. First, we investigated the action of gefitinib in a mouse model of acute peritoneal challenge with LPS followed by gefitinib, and found that gefitinib elevates the levels of mature-IL-1β in peritoneal lavage, which was clearly suppressed by MCC950 (Fig. 7A). Therefore, we next examined whether gefitinib elevates the levels of HMGB1 in peritoneal lavage. Since it has been reported that peritoneal challenge with LPS promotes HMGB1 release, we challenged gefitinib into peritoneal cavity without LPS, a condition that lacks the priming of peritoneal macrophages for pro-IL-1β induction. As shown in Fig. 7B, we observed HMGB1 in the peritoneal lavage of all gefitinib-challenged mice, and found that MCC950 fails to suppress HMGB1 release. These observations in vivo are completely consistent with the cell-based experiments, suggesting that gefitinib initiates mature-IL-1β and HMGB1 release via distinct mechanisms in vivo. It is known that interstitial pneumonitis is a most serious side effect induced by gefitinib. We thereby examined the potential mechanisms by which gefitinib causes interstitial pneumonitis. Since alveolar macrophages are mainly involved in the induction of lung inflammation, we investigated the response of alveolar macrophages to gefitinib ex vivo. As shown in Fig. 7C, alveolar macrophages isolated from bronchoalveolar lavage fluid (BALF) promoted NLRP3-dependent mature-IL-1β release in response to gefitinib, as well as BMDMs and THP-1 cells. Finally, we investigated the involvement of NLRP3 in lung inflammation induced by gefitinib. When murine lung was exposed to gefitinib via oropharyngeal aspiration (OPA), hematoxylin-eosin (HE) staining revealed that gefitinib initiates lung infiltration of inflammatory cells, and causes lung injury, which is suppressed by MCC950 treatment (Fig. 7D). Thus, our findings demonstrate that the NLRP3 inflammasome plays a key role in the induction of lung inflammation such as interstitial pneumonitis triggered by gefitinib.

## Discussion

**Fig. 8:**
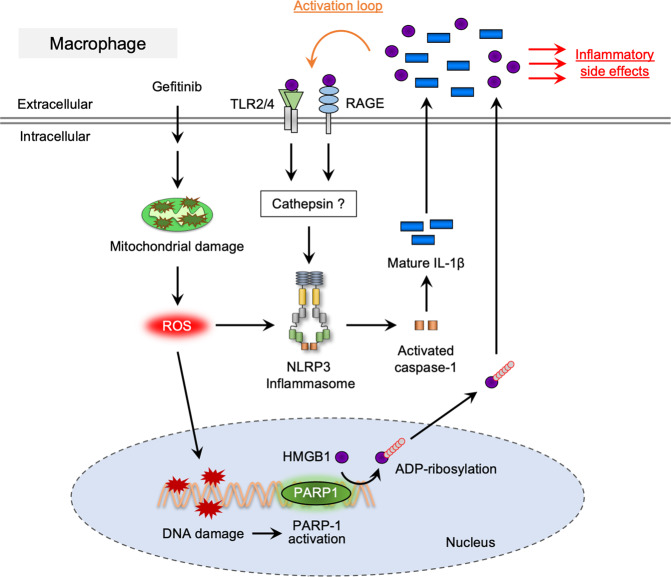
A proposed model for gefitinib-mediated necroinflammation. See the discussion section.

In Fig. 8, a schematic model to explain our study was visualized. We firstly noticed that gefitinib stimulates mature-IL-1β release in macrophages, and found that the NLRP3 inflammasome mediates gefitinib-induced mature-IL-1β release (Figs. 1 and 2). Although precise mechanisms of the NLRP3 inflammasome activation are still not fully understood, our results indicate that mtROS associated with mitochondrial damage driven by gefitinib are most likely to trigger the NLRP3 inflammasome activation (Fig. 3). To date, several pharmaceuticals have been reported to stimulate the NLRP3 inflammasome in a ROS-dependent manner^48–50^. In particular, imiquimod, a prescription medication used to treat genital warts, stimulates mtROS generation by inhibiting the quinone oxidoreductases NQO2 and mitochondrial Complex I, which triggers the NLRP3 inflammasome activation^50^. On the other hand, statins, the HMG-CoA reductase inhibitors commonly prescribed as cholesterol-lowering drugs, activate the NLRP3 inflammasome both transcriptionally and post-transcriptionally^51–53^. These findings imply that a substantial number of pharmaceuticals has the potential ability to stimulates the NLRP3 inflammasome activation. Although gefitinib was designed to specifically block the tyrosine kinase activity of EGFR, emerging evidence has demonstrated that gefitinib affects the activities of signaling molecules other than EGFR^25,54–56^. Notably, gefitinib has been reported to inhibit tyrosine kinase activity of receptor-interacting protein 2 (RIP2) and subsequent cytokine release^25^. Therefore, there is a possibility that gefitinib causes mitochondrial damage triggering the activation of the NLRP3 inflammasome through the off-target activity to specific tyrosine kinases.

We also found that gefitinib stimulates HMGB1 release (Fig. 5). Gefitinib-induced HMGB1 release was not affected by ASC knockout (Fig. 5D); however, ASC knockout THP-1 cells exhibited significant resistance to gefitinib-induced cell death (Fig. 4C). These observations indicate that gefitinib-induced HMGB1 release is caused by not passive leakage from dead cells but mechanisms that promote positive release of HMGB1. Moreover, MCC950 failed to inhibit gefitinib-induced HMGB1 release, whereas release of mature-IL-1β was strongly inhibited (Fig. 5C). Thus, the NLRP3 inflammasome activation and subsequent pyroptosis may not be required for the positive release of HMGB1 induced by gefitinib. On the other hand, nigericin-induced HMGB1 release completely depends on ASC, suggesting that NLRP3 inflammasome activation and subsequent pyroptosis are essential for the release (Fig. 5E). In addition, dependency of nigericin-induced cell death on pyroptosis appears to be much higher than that of gefitinib (Fig. 4A). Accordingly, nigericin-induced inflammation and death is largely governed by the NLRP3 inflammasome. However, there is no evidence in this study that the NLRP3 inflammasome positively stimulate HMGB1 release, and thus a possibility that nigericin-induced HMGB1 release is mediated by passive release from pyroptotic cells. In any case, to explain the stimulus-specific requirement of the NLRP3 inflammasome for HMGB1 release, further research is needed to investigate whether positive release of HMGB1 is promoted by the NLRP3 inflammasome.

Previous reports have demonstrated that PARP-1 mediates positive release of HMGB1, which is why we examined the involvement of PARP-1 in gefitinib-induced HMGB1 release^11,12^. As expected, both pharmacological and genetic inhibition of PARP-1 activation revealed that PARP-1 is required for gefitinib-induced HMGB1 release (Fig. 6G, I). Moreover, the antioxidant BHA completely inhibited both the accumulation of γH2AX and pADPr, suggesting that oxidative stress-induced DNA damage mediates PARP-1 activation (Fig. 6B, E). Notably, oxidative stress-induced PARP-1 activation is known to induce cell death accompanied by DNA fragmentation, which is specially designated parthanatos^31,57^. Unlike pyroptosis, parthanatos is not defined as inflammatory cell death, and so far the involvement of parthanatos in the HMGB1 release has not been investigated. We therefore tested whether parthanatos is involved in gefitinib-induced HMGB1 release. As shown in Fig. 6J, K, inhibition of the PARP-1 pathway failed to suppress gefitinib-induced cell death, indicating that contribution of parthanatos to gefitinib-induced cell death is relatively small. Therefore, we concluded that gefitinib-induced HMGB1 release is independent of parthanatos.

Gefitinib causes rare but fatal acute interstitial pneumonitis^23^. Our in vivo studies demonstrated that gefitinib administration via oropharyngeal aspiration caused lung inflammation, which was inhibited by MCC950 (Fig. 7D), suggesting the involvement of the NLRP3 inflammasome in gefitinib-induced interstitial pneumonitis. The risk factors for gefitinib-induced interstitial pneumonitis, smoking habit, pre-existing lung diseases, and poor performance status are statistically enumerated^58,59^. These risk factors are closely associated with the pathogenesis of mitochondrial dysfunction, and thus may increase the sensitivity to gefitinib-induced mitochondrial damage. A case report has shown that the impression of gefitinib-induced interstitial pneumonitis was reversed by treatment with methylprednisolone^60^. Interestingly, methylprednisolone can protect the rats from acute lung injury mediated by the NLRP3 inflammasome^61^. These findings support our model that gefitinib initiates lung inflammation through the NLRP3 inflammasome. On the other hand, our experimental conditions (10–20 µM gefitinib) are commonly used in cellular experiment using cancer cells^24,62^, whereas these dosages are much higher than clinical conditions (up to ~1.4 µM)^63^. However, in some cases, the concentration of gefitinib in breast tumor tissue increased up to 43-fold compared with plasma^63^. Therefore, the biased distribution of gefitinib may elicit focal activation of the macrophages. In addition, there are racial and ethnic differences in the frequency of gefitinib-induced interstitial pneumonitis^64,65^. In particular, the incidence of interstitial pneumonitis in the Japanese population has been reported to be higher than that in other populations^64,65^. However, our working model cannot explain these racial and ethnic differences, so further studies are needed to elucidate the reason.

As described in Fig. 8, it has been reported that HMGB1 release initiates a positive feedback loop of the NLRP3 inflammasome signaling, leading to further release of mature-IL-1β that is responsible for excessive inflammation^41^. The pharmacological properties of therapeutic drugs that promote HMGB1 release may increase the risk of inflammation-based side effects. Therefore, to uncover the mechanisms by which HMGB1 release is promoted will contribute to a better understanding of the pathogenesis of inflammatory diseases associated with the NLRP3 inflammasome.

## Materials and methods

### Cell culture

THP-1 cells were cultured in RPMI 1640 containing 10% heat-inactivated fetal bovine serum (FBS), 1% penicillin–streptomycin solution, and Plasmocin at 37 °C under a 5% CO_2_ atmosphere. For experiments, THP-1 cells were differentiated for 3 h with 100 nM PMA on the day before stimulation. Bone marrow-derived macrophages (BMDMs) were isolated from mouse femurs in sterile RPMI 1640 and were cultured in RPMI 1640 containing 10 ng/ml M-CSF and 10% heat-inactivated fetal bovine serum, and 1% penicillin–streptomycin solution in 5% CO_2_ at 37 °C atmosphere.

### Generation of KO cells

*NLRP3*, *ASC*, *caspase-1*, and *caspase-3* knockout cells were generated using the CRISPR/Cas9 system as previously described^66,67^. Guide RNAs (gRNAs) were designed to target a region in the exon 1 of *NLRP3* gene (5ʹ-CTGCAAGCTGGCCAGGTACC-3ʹ), the exon 1 of *ASC* gene (5ʹ-CAGCACGTTAGCGGTGAGCT-3ʹ), the exon 2 of *caspase-1* gene (5ʹ-AAGCTGTTTATCCGTTCCAT-3ʹ), and the exon 5 of *caspase-3* gene (5ʹ-CATACATGGAAGCGAATCAATGG-3ʹ) using CRISPRdirect. gRNA-encoding oligonucleotide was cloned into lentiCRISPRv2 plasmid (addgene), and knockout cells were established as previously described^66,67^. To determine the mutations of each gene in cloned cells, genomic sequence around the target region was analyzed by PCR-direct sequencing using extracted DNA from each clone as a template and the following primers: 5ʹ-AGTGTGGACCGAAGCCTAAG-3ʹ and 5ʹ-TTCTCCTCCCCATTGAAGTC-3ʹ for *NLRP3*; 5ʹ-TTGGACCTCACCGACAAG-3ʹ and 5ʹ-GCAGCTTTGTTTAGGGGTAGG-3ʹ for *ASC*; 5ʹ-TACCCAACTGTGAGGAGGGG-3ʹ and 5ʹ-TGGCCCTGAAGCCAGAAATAA-3ʹ for *caspase-1*, 5ʹ-GCAGCTTTGTTTAGGGGTAGG-3ʹ for *ASC*; 5ʹ- ACAGAAGGCGTGGTCATTTC-3ʹ and 5ʹ-TGTAGGTCCTGCCCAATCTC-3ʹ for *caspase-3*.

### Generation of PARP-1-knockdown cells

PARP-1 (MISSON shRNA, Sigma) or control shRNA (pLKO.1) lentivirus vector were co-transfected with the VSV-G envelope and psPAX2 packaging plasmids (Addgene) into human embryonic kidney (HEK) 293T cells using Lipofectamine 2000 (Thermo Scientific). After 48 h, lentivirus-containing supernatants were harvested and centrifuged at 1000 × *g* for 5 min to discard the debris. THP-1 cells were infected with the virus for 24 h and then selected by 1 µg/ml puromycin for 72 h. Knockdown efficacy was analyzed by immunoblotting.

### Reagents and antibodies

All reagents were obtained from commercial sources; Gefitinib, BHA, JC-1 (Wako), PMA, Mito-TEMPO, DPQ, nigericin (Santa Cruz), disuccinimidyl suberate (DSS), MitoSOX Red (Thermo Scientific), Glyburide (Sigma), Z-VAD-fmk (Peptide Institute), MCC950 (Cayman and Adipogen). The antibodies used were against NLRP3 (Adipogen), caspase-3, IL-1β (Cell Signaling), ASC (MBL), caspase-1, HMGB1, PARP-1, pADPr, γH2AX, Tom20, and β-actin (Santa Cruz).

### Immunoblotting

Proteins from cell culture supernatants were extracted by methanol/chloroform precipitation. In brief, cell-free supernatant was mixed with methanol:chloroform at a 5:5:1 (cell culture supernatant/methanol/chloroform) ratio. The mixture was vortexed and centrifuged for 10 min at 15,000 rpm. The clear upper phase was discarded, and 1000 μl methanol was added to the interphase. The mixture was centrifuged for 10 min at 15,000 rpm, and the liquid phase was removed. The protein pellet was dried, resuspended with 8 M Urea. Cells were lysed in ice-cold lysis buffer containing 20 mM Tris-HCl (pH 7.4), 150 mM NaCl, 1% Triton X-100, 10% glycerol, and 1% protease and phosphatase inhibitor mixtures (Nacalai Tesque). Samples were resolved by sodium dodecyl sulfate polyacrylamide gel electrophoresis and analyzed as described previously^68^.

### ASC oligomerisation assay

ASC oligomerisation assay was performed as described previously with minor modifications^28^. Briefly, the cells were washed with phosphate-buffered saline (PBS) and harvested in buffer A (20 mM HEPES-KOH (pH 7.5), 10 mM KCl, 1.5 mM MgCl_2_, 1 mM EDTA, 1 mM EGTA, 320 mM sucrose, 1% protease inhibitor cocktail). The cells were lysed by shearing 15 times through a 27-gauge needle, and then the cell lysates were centrifuged at 600 × *g* to remove the bulk nuclei and unbroken cells. The supernatants were centrifuged at 17,700 × *g*, and then the pellets including ASC oligomer were resuspended in CHAPS buffer (20 mM HEPES-KOH (pH 7.5), 5 mM MgCl_2_, 0.5 mM EGTA, 0.1% CHAPS, 1% protease inhibitor cocktail) and were reacted with 1.5 mM disuccinimidyl suberate (DSS) for 30 min.

### Enzyme-linked immunosorbent assay (ELISA)

Concentrations of IL-1β and TNF-α in cell culture supernatants were measured by specific ELISA kits (IL-1β; Thermo Fisher Scientific; TNF-α; BD Biosciences) according to the manufacturer’s instructions.

### Cell death assay

Cell death was monitored by using LDH-Cytotoxic Test Kit (Wako) according to the manufacturer’s protocol. The activity level of the LDH released into the culture media was quantified as a percentage of the total activity level of LDH as described previously^69^.

### Bioimaging and quantification of ROS

THP-1 cells were seeded on glass bottom dishes. After stimulation, cells were treated with 1 μM MitoSOX for 30 min at 37 °C. After washing with PBS, the intracellular ROS generation was observed using a Zeiss LSM800 laser confocal microscope (Carl Zeiss) and the images were processed with Zen software. The fluorescence images were obtained from three different fields of view as described previously^31^. Data shown are the mean ± SD of three images.

### Quantitative real-time PCR

Total RNA was extracted using Sepasol-RNA I Super G (Nacalai Tesque) and reverse transcribed using High-Capacity cDNA Reverse Transcription Kit (Applied Biosystems) according to the manufacturer’s instructions. Template cDNA was amplified by quantitative real-time PCR as described previously^24^. Primers used for qRT-PCR; 5ʹ-ATAGAATTCATGGCAGAAGTACCTGAGCT-3ʹ and 5ʹ-TATCTCGAGTTAGGAAGACACAAATTGCA-3ʹ for *pro-IL-1β*, and 5ʹ-AACAGCCTCAAGATCATCAGC-3ʹ and 5ʹ-GGATGATGTTCTGGAGAGCC-3ʹ for *GAPDH*. Each gene expression levels were normalized to that of *GAPDH*.

### Animal experiments

C57BL/6J mice were purchased from CLEA Japan. The mice were maintained according to the Guidelines for Animal Experimentation of Tohoku University, and all the procedures were approved by the Institutional Animal Care and Use Committee at Tohoku University (approval number: 2020PhA-017). For IL-1β determination, male C57BL/6 (6-8 weeks) mice were treated with 1 µg of LPS and 20 mg/kg MCC950 in PBS by intraperitoneal injection. After 2 h, mice were treated with 10 mg/kg gefitinib and 20 mg/kg MCC950 in 0.5 ml of PBS/dimethyl sulfoxide **(**DMSO) by intraperitoneal injection. After 0.5 h, peritoneal cavities were lavaged with ice-cold PBS, and then heparin and protease inhibitor cocktail were added to the peritoneal lavage fluid. Collected peritoneal lavage fluid was centrifuged for 3 min at 5000 rpm to separate fluid from cells. IL-1β levels in the supernatants were analyzed by ELISA. For HMGB1 determination, C57BL/6 (6-8 weeks) mice were treated with 25 mg/kg MCC950 in PBS/DMSO by intraperitoneal injection 0.5 h before and after intraperitoneal injection 10 mg/kg gefitinib in PBS/DMSO containing 0.05% Tween-80. After 4 h, peritoneal cavities were lavaged with ice-cold PBS. Collected peritoneal lavage fluid was centrifuged for 3 min at 5000 rpm to separate fluid from cells. 1% Triton X-100 were added to the supernatants, and then peritoneal lavage fluid were subjected to immunoblotting with anti-HMGB1 antibody. For histological analysis of lung tissue, C57BL/6 (6–8 weeks) mice were treated 20 mg/kg gefitinib and 2.5 mg/kg MCC950 in PBS/DMSO containing 0.05% Tween-80 by oropharyngeal aspiration. After 2 days mice were euthanized, and then the left lungs were histologically analyzed.

### Primary alveolar macrophages isolation

Primary alveolar macrophages were isolated from the lungs of mice by bronchoalveolar lavage (BAL) with 3–4 washes of 1 ml ice-cold PBS containing 100 µM EDTA. Collected BAL fluid was centrifuged for 3 min at 1000 rpm to separate fluid from cells. The cell pellet was resuspended with RPMI 1640 containing 10% FBS. Cells were seeded in collagen coated 48-well plates and were allowed to adhere for 1–3 h. Non-adherent cells were removed by washing with PBS.

### Histological analysis

Left lungs of mice were fixed with 4% paraformaldehyde in PBS. Tissues were embedded in paraffin, sectioned (7 μm), and stained with hematoxylin-eosin (HE). Stained tissues were photographed under a light microscope (Zeiss AxioVision). Lung tissue was evaluated by applying an arbitrary grading scale ranging from 0 to 8 (0 = no changes, 1 = minimal lesions affecting 1-10% of the area, 2 = lesions affecting 10–20% of the area, 3 = changes affecting 20–30% of the area, 4 = changes affecting 30–40% of the area, 5 = changes affecting 40–50%, 6 = changes affecting 50–60%, 7 = changes affecting 60–70%, and 8 = changes affecting >80% of the area). The following affected pulmonary parameters were evaluated: alveolar edema, alveolar hemorrhage, interstitial cellular infiltrates and edema, perivascular cellular infiltrates and edema, and alveolar epithelial necrosis.

### Statistical analysis

The value was expressed as the mean ± standard deviation (S.D.) using Prism software (GraphPad). All experiments were repeated at least three independent times. Two groups were compared using student’s *t*-test. Multiple-group comparisons were conducted using the one-way ANOVA analysis of variance followed by the Tukey–Kramer test using Prism software (GraphPad). Data were considered significant when *p < 0.05, **p < 0.01, ***p < 0.001.
